# Legislating for public accountability in universal health coverage, Thailand

**DOI:** 10.2471/BLT.19.239335

**Published:** 2019-12-04

**Authors:** Kanang Kantamaturapoj, Anond Kulthanmanusorn, Woranan Witthayapipopsakul, Shaheda Viriyathorn, Walaiporn Patcharanarumol, Churnrurtai Kanchanachitra, Suwit Wibulpolprasert, Viroj Tangcharoensathien

**Affiliations:** a Faculty of Social Sciences and Humanities, Mahidol University, Nakhon Pathom, Thailand.; b International Health Policy Program, Ministry of Public Health, Tiwanond Road, Nonthaburi, Thailand 11000.; c Institute for Population and Social Research, Mahidol University, Nakhon Pathom, Thailand.

## Abstract

Sustaining universal health coverage requires robust active public participation in policy formation and governance. Thailand’s universal coverage scheme was implemented nationwide in 2002, allowing Thailand to achieve full population coverage through three public health insurance schemes and to demonstrate improved health outcomes. Although Thailand’s position on the World Bank worldwide governance indicators has deteriorated since 1996, provisions for voice and accountability were embedded in the legislation and design of the universal coverage scheme. We discuss how legislation related to citizens’ rights and government accountability has been implemented. Thailand’s constitution allowed citizens to submit a draft bill in which provisions on voice and accountability were successfully embedded in the legislative texts and adopted into law. The legislation mandates registration of beneficiaries, a 24/7 helpline, annual public hearings and no-fault financial assistance for patients who have experienced adverse events. Ensuring the right to health services, and that citizens’ voices are heard and action taken, requires the institutional capacity to implement legislation. For example, Thailand needed the capacity to register 47 million people and match them with the health-care provider network in the district where they live, and to re-register members who move out of their districts. Annual public hearings need to be inclusive of citizens, health-care providers, civil society organizations and stakeholders such as local governments and patient groups. Subsequent policy and management responses are important for building trust in the process and citizens’ ownership of the scheme. Annual public reporting of outcomes and performance of the scheme fosters transparency and increases citizens’ trust.

## Résumé

Maintenir la couverture sanitaire universelle exige une forte participation publique à l'élaboration des politiques et à la gouvernance. En Thaïlande, le régime de couverture universelle a été mis en œuvre dans tout le pays en 2002, permettant de couvrir l'ensemble de la population grâce à trois régimes publics d'assurance maladie et d'améliorer les résultats de santé. Bien que la position de la Thaïlande concernant les Indicateurs de gouvernance mondiaux de la Banque mondiale se soit détériorée depuis 1996, des dispositions en matière d'expression et de reddition de comptes ont été intégrées à la législation et à la structure du régime de couverture universelle. Nous discutons ici de la mise en œuvre de la législation relative aux droits des citoyens et à la reddition de comptes du gouvernement. En vertu de la constitution de la Thaïlande, les citoyens ont pu soumettre un projet de loi dont les dispositions en matière d'expression et de reddition de comptes ont été intégrées aux textes législatifs et transposées dans la loi. La législation rend obligatoire l'enregistrement des bénéficiaires, une assistance téléphonique 24h/24 et 7 j/7, des auditions publiques annuelles et une aide financière systématique pour les patients qui ont été victimes d'événements indésirables. Pour garantir le droit à des services de santé, permettre aux citoyens de faire entendre leur voix et s'assurer que des mesures soient prises, les institutions doivent être en mesure d’appliquer la législation. Par exemple, la Thaïlande devait pouvoir enregistrer 47 millions de personnes et les rattacher au réseau de prestataires de soins du district où elles vivaient, et réenregistrer les personnes qui changeaient de district. Les auditions publiques annuelles doivent faire participer les citoyens, les prestataires de soins, les organisations de la société civile et les parties prenantes telles que les collectivités locales et les groupes de patients. Les réponses qui en découlent au point de vue des politiques et de la gestion sont importantes pour instaurer la confiance dans le processus et permettre aux citoyens de se l'approprier. Les rapports annuels publics sur les résultats du régime de couverture permettent d'accroître la transparence et de renforcer la confiance des citoyens.

## Resumen

Para mantener la cobertura sanitaria universal se requiere una sólida participación activa del público en la formulación de políticas y la gobernanza. El plan de cobertura universal de Tailandia se implementó en todo el país en 2002, lo que permitió a Tailandia lograr una cobertura completa de la población a través de tres planes de seguro médico público y demostrar mejores resultados en materia de salud. Aunque la posición de Tailandia respecto de los Indicadores mundiales de gobernanza del Banco Mundial ha disminuido desde 1996, las disposiciones relativas a la voz y la rendición de cuentas estaban incorporadas en la legislación y en el diseño del plan de cobertura universal. Se discute cómo se ha implementado la legislación relacionada con los derechos de los ciudadanos y la rendición de cuentas del gobierno. La Constitución de Tailandia permitía a los ciudadanos presentar un proyecto de ley en el que las disposiciones sobre la voz y la rendición de cuentas se incorporaban con éxito en los textos legislativos y se aprobaban como ley. La legislación exige el registro de los beneficiarios, una línea telefónica de ayuda 24 horas al día los 7 días de la semana, audiencias públicas anuales y asistencia financiera gratuita para los pacientes que han sufrido eventos adversos. Para garantizar el derecho a los servicios de salud y que se escuche la voz de los ciudadanos y se adopten medidas, es necesario contar con la capacidad institucional para aplicar la legislación. Por ejemplo, Tailandia necesitaba la capacidad de inscribir a 47 millones de personas y ponerlas en contacto con la red de proveedores de servicios de salud del distrito en el que viven, y de volver a inscribir a los miembros que se trasladan fuera de sus distritos. Las audiencias públicas anuales deben incluir a los ciudadanos, los proveedores de servicios de salud, las organizaciones de la sociedad civil y las partes interesadas, como los gobiernos locales y los grupos de pacientes. Las respuestas políticas y de gestión subsiguientes son importantes para generar confianza en el proceso y en la apropiación del plan por parte de los ciudadanos. El informe público anual sobre los resultados y el rendimiento del plan fomenta la transparencia y aumenta la confianza de los ciudadanos.

## ملخص

يتطلب دعم التغطية الصحية الشاملة مشاركة نشطة وقوية في وضع السياسات والحوكمة. تم تنفيذ خطة التغطية الشاملة في تايلند على مستوى البلاد في عام 2002، مما سمح لتايلند بتحقيق التغطية الكاملة لسكانها من خلال ثلاث خطط للتأمين الصحي الشعبي، واستعراض النتائج الصحية المُحسنة. على الرغم من أن موقف تايلاند وفقا لمؤشرات البنك الدولي للحوكمة العالمية قد تدهور منذ عام 1996، إلا أنه قد تم تضمين أحكام للمساءلة والتعبير عن الرأي في التشريعات، وكذلك في وضع خطة التغطية الشاملة. نحن نناقش كيف تم تنفيذ التشريعات المتعلقة بحقوق المواطنين ومساءلة الحكومة. سمح الدستور التايلاندي للمواطنين بتقديم مشروع قانون، حيث تم في هذا القانون بنجاح تضمين فقرات للمساءلة وحرية الرأي في النصوص التشريعية، وأُضيفت في القانون. ينص التشريع على تسجيل المستفيدين، ووجود خط المساعدة على طوال اليوم على مدار الأسبوع، وكذلك جلسات استماع عامة سنوية، والمساعدة المالية لتعويض المرضى الذين عانوا من أحداث سلبية. إن ضمان الحق في الحصول على الخدمات الصحية، والاستمتاع لآراء المواطنين واتخاذ الإجراءات اللازمة، يتطلب القدرة المؤسسية على تنفيذ التشريعات. على سبيل المثال، احتاجت تايلند إلى القدرة على تسجيل 47 مليون شخص، ومطابقتهم مع شبكة مقدمي الرعاية الصحية في المنطقة التي يعيشون فيها، وإعادة تسجيل الأعضاء الذين ينتقلون خارج مناطقهم. يجب أن تشتمل جلسات الاستماع العامة السنوية على المواطنين، ومقدمي الرعاية الصحية، ومنظمات المجتمع المدني، وأصحاب المصلحة مثل الحكومات المحلية ومجموعات المرضى. تعد الاستجابات التالية للسياسة والإدارة هامة لبناء الثقة في العملية، وفي ملكية المواطنين للخطة. إن التقارير العامة السنوية حول النتائج وأداء الخطة، تعزز الشفافية وتزيد من ثقة المواطنين.

## 摘要

如需维持全民健康覆盖，就需要公众积极参与政策制定和治理。泰国计划于 2002 年在全国范围内实施全民健康覆盖，这将使得泰国通过三项公共健康保险计划实现全民覆盖，并有效改善公民健康状况。尽管自 1996 年以来，泰国在世界银行《全球治理指数》中的地位有所下降，但有关发言权和问责制的条款已纳入全民覆盖计划的立法和设计之中。我们探讨与公民权利和政府问责相关的法规是如何实施的。泰国宪法允许公民提交一项草案，其中关于发言权和问责制的条款已成功地纳入法规案文成为法律条文。该立法要求对受益人进行登记，开通一条 24/7 全天候服务的求助热线，每年举行公众听证会，并为经历过不良事件的患者提供无过错财政援助。相关执法机构需要有能力确保公民享有健康服务的权利，倾听公民的声音并采取行动。例如，泰国需要有能力对 4700 万人口进行登记，并将他们与居住地区的医疗护理提供者的网络相匹配，并对迁出其居住地区的成员进行重新登记。每年的公众听证会必须包括公民、医疗护理提供者、民间社会组织和利益相关者，如地方政府和患者团体。后续政策和管理对策的好坏将直接影响公民是否信任全民健康覆盖和公民对该计划的所有权。每年公开报告该计划的成果和执行情况有助于提高透明度，提高公民的信任感。

## Резюме

Поддержание всеобщего охвата услугами здравоохранения требует активного участия общественности в формировании политики и организации управления. Схема всеобщего охвата услугами здравоохранения Таиланда была внедрена на всей территории страны в 2002 году, что позволило Таиланду добиться полного охвата населения услугами здравоохранения с помощью трех схем государственного медицинского страхования и продемонстрировать улучшение результатов мероприятий по охране здоровья. Несмотря на то что позиция Таиланда по Всемирным индикаторам управления Всемирного банка ухудшается с 1996 года, положения о праве голоса и подотчетности были включены в законодательство и схему всеобщего охвата услугами здравоохранения. Авторы обсуждают вопросы реализации законодательства, касающиеся прав граждан и подотчетности правительства. Конституция Таиланда позволяет гражданам вносить законопроекты, с помощью которых положения о праве голоса и подотчетности были успешно включены в законодательные тексты и приняты в закон. Законодательство предписывает регистрацию бенефициаров, обеспечение круглосуточной работы телефонной линии помощи, проведение ежегодных общественных слушаний и оказание финансовой помощи пациентам, испытавшим нежелательные явления, независимо от причин, по которым это произошло. Обеспечение права на медицинское обслуживание, а также учета мнения граждан и принятия соответствующих мер требует наличия организационного потенциала для исполнения законодательства. Например, Таиланду нужны были ресурсы, которые позволили бы зарегистрировать 47 миллионов человек и сопоставить их данные с сетью поставщиков медицинских услуг в районе их проживания, а также перерегистрировать участников, меняющих район проживания. В ежегодных общественных слушаниях должны участвовать представители от граждан, поставщиков медицинских услуг, общественных организаций и заинтересованных сторон, таких как местные органы власти и группы пациентов. Последующие политические и управленческие меры реагирования важны для укрепления доверия общественности к процессу и повышения реальной заинтересованности граждан в этой схеме. Ежегодная общедоступная отчетность по результатам мероприятий по охране здоровья и эффективности работы схемы способствует обеспечению прозрачности процесса и повышает доверие граждан.

## Introduction

**Fig. 1:**
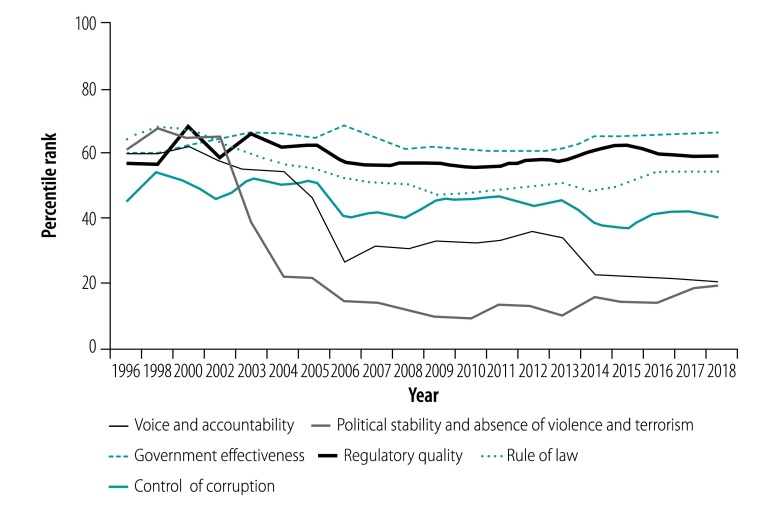
Percentile rank of worldwide governance indicators, Thailand 1996–2018

The World Bank worldwide governance indicators^1^ comprise six dimensions of governance: voice and accountability; political stability and absence of violence; government effectiveness; regulatory quality; rule of law; and control of corruption. The indicators relate to national level governance, and none are specifically about health. The voice and accountability indicator “captures perceptions of the extent to which a country’s citizens are able to participate in selecting their government, as well as freedom of expression, freedom of association and a free media.”^2^ Between 1996 and 2018, Thailand’s overall ranking on the indicators deteriorated, affected by the country’s protracted political conflicts since 2002.^3^ From 2002 to 2018, Thailand’s global rank has decreased from the 65th to below the 20th percentile for political stability and from the 60th to the 20th percentile for voice and accountability. However, government effectiveness remained relatively stable around the 60th and 70th percentiles (Fig. 1). Public services remain functioning with adequate quality, reflecting a degree of independence from political pressure and a capacity to formulate and implement policies among bureaucrats.

Data source: World Bank worldwide governance indicators.^4^

Sustaining universal health coverage (UHC) requires robust active public participation^5^ in policy formation and accountability mechanisms.^6^^–^^8^ Participatory governance can improve the performance of the health system.^9^ Partnerships and opportunities for dialogue among multiple stakeholders are therefore important for health-sector governance. In New Zealand, Thailand and Turkey, accountability mechanisms have been shown to support quality and responsiveness of services through ensuring that health professionals respect patients’ rights.^10^^,^^11^

Since 2002, Thailand’s entire population of 63 million has been entitled to a comprehensive health benefit package with a high level of financial risk protection through one of the three public insurance schemes. The civil servant medical benefit scheme for government employees, pensioners and dependents (spouse, parents and not more than three children younger than 20 years) is managed by the Comptroller General’s department of the finance ministry. The social health insurance for private sector employees is managed by the Social Security Office of the labour ministry. The remaining population are covered by the universal coverage scheme, managed by the National Health Security Office, a public body established under the National Health Security Act 2002.^12^

Since its introduction, the universal coverage scheme has contributed to favourable health outcomes. Access to health services by the whole population has improved, with low levels of unmet health care needs,^13^ comparable to Organisation for Economic Co-operation and Development countries.^14^ Outpatient and inpatient utilization of public health-care facilities has increased, preferentially benefitting elderly people.^15^ Use of annual check-ups has increased, particularly among women,^16^ with no evidence of greater consumption of health-care services. The scheme benefits poor households, who are more likely to use public health services than richer people, with pro-poor budget subsidies and services requiring no copayments.^17^ Extensive geographical coverage by well-functioning district health systems, developed since before the introduction of the scheme, explains the pro-poor outcomes.^18^

In this article we identify the provisions on voice and accountability in Thailand’s legislation on UHC and consider how the universal coverage scheme is designed to ensure citizen’s voices and concerns are heard and taken into consideration. The deliberative process in the scheme provides lessons for low- and middle-income countries and other sectors in Thailand where policy links are weak, such as education, environment and social welfare.

## Legislation

Article 56 of the 2017 Constitution of Thailand requires the government to conduct public hearings and environmental and health impact assessments^19^ for policies which may have a negative impact on culture, health and the environment.^20^ The National Health Security Act, however, set up additional processes which foster implementation of voice and accountability. Embedded in the Act are six articles related to citizens’ voices and the accountability of the National Health Security Office (Table 1). Article 18(10) and Article 18(13) mandate the office to convene annual public hearings for health-care providers and patients on the challenges faced and to identify gaps for improving the performance of the universal coverage scheme.^21^ Article 26(3) and Article 26(7) mandates the office to register citizens to health-care provider networks and record the information in the national beneficiary database and re-register members to a new network if they relocate. Articles 26(8), 50(5), 57 and 59 further mandate the office to establish systems for citizens to lodge complaints and for conflicts to be investigated and resolved.^21^ Article 41 mandates the office to earmark up to 1% of the total annual budget of the scheme for no-fault financial assistance to the patients or families affected by adverse events.^21^^,^^22^

**Table 1:** Voice and accountability provisions in Thailand’s National Health Security Act 2002 and actions taken

Related articles in the National Health Security Act 2002	Corresponding actions by the National Health Security Office	Implications
**Article 18(10)**: the National Health Security Board shall prescribe rules for hearing opinions of providers and patients to improve the quality and standard of health services. **Article 18(13)**: the National Health Security Board has a duty to conduct annual general public hearings with health-care providers and patients	Annual general public hearings are conducted at regional and national levels	Key stakeholders in the universal coverage scheme, including health-care providers and patients, have a channel to voice their concerns about the scheme. The board is responsible for improving the quality of health services based on the results of public hearings
**Article 26(3)**: the National Health Security Office is responsible for registration and update on the status of the universal coverage scheme members. **Article 26(7)**: a universal coverage scheme member can re-register with health-care networks, on request	A beneficiary registration system is publicly accessible via the office’s website. The system is updated monthly	The office is accountable for ensuring the accessibility of universal coverage scheme members to health-care units and ensuring uninterrupted rights to health services among people relocating for work
**Article 26(8)**: the National Health Security Office shall facilitate and manage citizens’ complaints. **Article 50(5)**: the National Health Security Office shall provide an independent complaint unit from health-care providers. **Article 57**: a health-care unit that fails to comply with the prescribed health service standard shall be investigated. **Article 59**: patients who are not provided with reasonable facilitation shall lodge their complaints to the National Health Security Office for investigation under Article 57	A telephone helpline provides information to patients, scheme members, as well as health workers about the universal coverage scheme and its benefit package, how to locate the required services and how to lodge complaints. Health security service centres in 885 hospitals deal with on-site problem-solving and helps patients to navigate through the health-care system. Civil society organizations manage community-based complaint units, independent from health-care providers	The office is accountable for protecting the rights of universal coverage scheme members to standard health services. Civil society organizations manage community-based complaint units, that are independent from health-care providers, ensure that members’ voices are heard and local action is taken
**Article 41**: the National Health Security Board shall earmark not more than 1% of the National Health Security Fund for initial financial assistance to patients affected by adverse events due to medical services	Initial financial assistance is provided to patients or families affected by an adverse event or death	The office is accountable for prompt responses to adverse events due to medical services

## From legislation to action

### Annual public hearings

The annual public hearings are an integral part of the universal coverage scheme since 2004 (the civil servant medical benefit scheme and social health insurance have no such mechanism). In implementing the legislative mandate, the National Health Security Office strives to create a sense of ownership among members of the scheme and gain broad-based support from other stakeholders.^23^ Engagement with health-care providers strengthens the scheme and ensures it benefits its members.^24^ Although public hearings for providers and beneficiaries are mandatory, the office also creates opportunities for other stakeholders, in particular representatives from local administrative organizations and academia, to express their views and provide recommendations.^25^ Regional health security offices request provincial health offices to nominate representatives of health-care providers. Provincial coordination centres, managed by civil society organizations, nominate lay people to attend the hearings and inform attendees about the process.^26^ To accommodate distinct interests and avoid possible conflict, provider and citizen hearings are convened separately. Reports on the public hearings and the management responses are circulated to affirm that the members’ voices were heard.

The office, as a conscious learning organization, has made several modifications to the public hearing process. In the first year, annual public hearings were trialled in the capital city Bangkok and four regions. They were later implemented in all 13 public health regions in 2005 and all provinces in 2006.^27^ In 2013, seven issues were identified for discussion at annual hearings: type and scope of essential health services; health service standards; office management; national health security fund management; local health security fund management; public participation; and other specific issues relevant to the locality.^28^ The opinions and suggestions from the 13 regional public hearings are compiled, synthesized and used as inputs for the final national level public hearing. All inputs and responses to proposals from the hearings are considered to identify further actions to be taken: a genuine and meaningful process demonstrating transparency and accountability.^25^

A few notable changes have stemmed from public hearings and the advocacy efforts of civil society organizations. Access to emergency health services was harmonized across the three public health insurance schemes in 2012, while in 2013 the criteria for no-fault financial assistance were revised. In 2015, the two-child limit on the number of birth deliveries eligible for the universal coverage scheme was abolished.^28^ Finally, stakeholders (policy-makers, medical experts, academia, research and innovation organizations, private industry, patient groups, civil society organizations and the general public) were able to participate in submissions of topics for consideration and the prioritization of new interventions included in the benefit package.^29^^–^^34^

### Registration of members

To ensure citizens’ rights to standard health care the National Health Security Office is mandated to register eligible members in the national beneficiary database and to update the database for births, deaths and movement across insurance schemes and health-care facilities. Citizens must be registered to a primary health-care contractor network in the district where they live and be re-registered to a new network if they relocate. As scheme members are required to use the network they are registered with, prompt re-registration for people seeking job opportunities away from their home district reflects the office’s accountability to protect members’ right to health services. The beneficiary registration system is publicly accessible via the office’s website and the system is updated monthly.

### Helpline

Since 2002 the National Health Security Office has managed a 24-hour, 7 days a week telephone helpline for people to obtain information about the universal coverage scheme and its benefit package, to locate the services they require and to lodge complaints. The Social Security Office also operates a 24/7 helpline, while the Comptroller General department’s call centre is only active during office hours.

Over the past two decades, the helpline service has evolved to make the universal coverage scheme more responsive to members’ needs and has analysed the data gathered to improve the scheme’s performance. Initially, only 10 staff members operated the call centre using a paper-based recording system. From 2004, record-keeping as well as information for call-centre workers was computer-based. A patient referral coordination service, facilitating referrals from one hospital to another, was incorporated in 2013. In 2018, Thai sign language services were introduced along with a telecommunication relay service, extending the service to 0.38 million beneficiaries with hearing disabilities, reflecting the office’s accountability to disabled users.^35^ By 2019, there were 78 full-time staff in the call centre, and an additional set of 21 staff managing complaints.

In 2018, 930 302 calls were received, of which 900 984 (96.8%) were enquiries about the benefit package, entitlements and co-payments, how to register for the health-care provider network and how to access health services. Complaints from patients accounted for 0.6% of the total calls (5248 complaints); 3672 of the 4531 resolved complaints (81.0%) were settled within 25 days, while 65 complaints (1.2%) were serious and submitted for investigation by the Quality and Standards Committee.^36^ A further 35 complaints concerned “health care units failing to meet the prescribed standard of service,” of which 13 were resolved by issuing an order advising health-care units to comply with the standard, three complaints were dismissed and 19 are under investigation. Another 30 complaints were about “health units not providing treatment pursuant to their rights or unduly charging the patients,” of which 11 complaints were resolved by requesting the health-care units to return money. Most complaints were resolved through communication and dialogue between providers and patients.

### No-fault financial assistance

Financial assistance for patients or families affected by adverse events, such as disability or death after using medical services, also reflects the high level of accountability in the universal coverage scheme. As mandated, the National Health Security Office earmarked 4.92 Thai baht (THB) per capita (United States dollars, US$ 0.16) for the 2018 fiscal year budget to no-fault financial assistance for adverse events, a total sum of 236.16 million THB (US$ 7.56 million). In 2018, 970 patients filed for the assistance and the Quality and Standards Committee, responsible for investigating and granting decisions, approved 755 (77.8%) patients to receive compensation, a total amount of 165.51 million THB (US$ 5.30 million).^36^ Additionally, 511 health professionals filed for compensation due to adverse events from providing services to patients, of whom 427 (83.6%) received compensation, totalling 6.31 million THB (US$ 0.21 million).^36^

Legislation under the universal coverage scheme has also influenced other government schemes. In 2018, the Social Security Office instituted a similar regulation to compensate social health insurance members for deaths, disability and conditions requiring long-term support. In the same year, the finance ministry has issued regulations to provide compensation to public health-care providers for adverse events, financed by the annual budget.^37^^,^^38^

## Governance and capacities

### Inclusiveness

The National Health Security Board directs and oversees the performance of the National Health Security Office. The multistakeholder nature of the Board is effective in ensuring accountability in decision-making and representing the views of the taxpaying public and beneficiaries of the universal coverage scheme. Board members include the health minister as chair, eight ex-officio members (permanent secretaries from the relevant ministries, including public health) four local government representatives, five civil society organization representatives, five health professionals including representatives from the private hospital association and seven experts in the fields of health insurance, medicine and public health, Thai traditional medicine, alternative medicine, health financing, law and social sciences. 

Representation by civil society organizations demonstrates the participation and empowerment of citizens. Organizations choose five from nine civil society organizations constituencies whose works are related to: children and adolescents; women; elderly people; disabled people and mentally ill patients; people living with human immunodeficiency virus and chronic diseases; labour issues; slum inhabitants; agriculture; and ethnic minorities. These constituencies reflect the broad-based representation of civil society organizations from throughout the country, whose strong advocacy on the board has helped expand the members’ benefit package.^39^ Another benefit is the greater continuity and institutional memory among civil society representatives than the eight ex-officio board members, owing to the rapid turnover of senior officials at the permanent secretary level. Although each term of office is only four years and civil society representatives are limited to two terms, new civil society representatives on the board always follow-up on issues of concern through their networks and maintain the continuity of their work in the board’s discussions.

Article 48 of the National Health Security Act established the Quality and Standards Committee, equivalent to the National Health Security Board. There are 39 committee members, including five civil society representatives, who oversee the quality and standard of health-care providers and approve no-fault financial assistance.

Public accountability and transparency are ensured through the provision in Article 18(12) of the law, which states that the board shall provide annual reports on performance and challenges, including audited financial reports to the Cabinet, the House of Representatives and the Senate within six months of the fiscal year end. There are no such provisions in the Social Security Act or in the Royal Decree of the Civil Servant Medical Benefit Scheme, despite both insurance schemes also being publicly financed. All National Health Security Office annual reports are made publicly available on the organization’s website and the board’s decisions have been published on its website since 2002.

### Institutional capacities

The National Health Security Office’s institutional capacity is crucial for ensuring citizens’ voices are heard and that office and health-care providers remain accountable to the citizens they serve. Without these capacities, the legislative provisions would be empty promises. In 2018, a total of 893 staff members worked across office headquarters and its 13 regional offices, of which about one-third had a health background.^36^ Almost all executive positions are held by experienced and highly qualified medical and health professionals.^23^ Unlike the Social Security Office which has two functions – collecting payroll tax and purchasing health services – the National Health Security Office’s only function is to purchase health services with additional efforts going into ensuring accountability to its members.

## Lessons learnt

Voice and accountability in Thailand’s universal coverage scheme is a deliberative process through which citizens’ voices are heard. The National Health Act 2007 mandates the convening of an annual national health assembly that provides a participatory platform for public policy development^40^ through multisectoral action.^41^ The assembly brings together three elements to effect change: evidence from the scientific community; civic movement by civil society organizations; and decision through political engagement.^42^ In Thailand this process is described as the triangle [of actions] that moves the mountain [of change]. Certain resolutions adopted by the assembly are endorsed by the Cabinet, giving implementing agencies within government the power to enforce them. On the other hand, the constitutional mandate for government agencies to conduct public hearings and environmental and health impact assessments is inadequate for responding to the concerns raised and challenges identified. This challenge undermines the objectives of public hearings^43^ and future participation in environmental and health impact assessments.^44^

We have identified two main factors, which we believe facilitated the effectiveness of voice and accountability in universal coverage scheme governance: legislative provisions and the deliberative process.

### Legislative provisions

The provisions in legislative documents are important because they legitimize all concerned agencies to implement the law. In the case of voice and accountability, it was the citizens themselves, through civil society organizations, who led the insertion of these provisions into the National Health Security Act 2002 to ensure that their voice would be heard once the Act was signed into law. Historically, Article 170 of the 1997 Constitution of Thailand^45^ allows 50 000 eligible voters to submit a draft bill for consideration by the National Legislative Assembly. The citizen-led draft UHC bill in 2002 was the first action to test this constitutional right. Through the efforts of civic groups, over 50 000 signatures were collected and the bill was submitted.^24^^,^^46^ Six competing draft universal coverage scheme bills were proposed to the government, one by the cabinet, four by political parties and one by citizen groups. After the first reading, which accepted the draft bill in principle, members of civic groups were appointed to the parliamentary committee to consider the second reading (article by article) and the third reading, which endorsed the final text. The key items of each draft bill were negotiated and eventually finalized as the National Health Security Act 2002.^47^ Key provisions proposed by citizens in the draft bill, particularly in relation to accountability and voice, were included in the final text endorsed by the House of Representatives and the Senate. However, legislative provisions, although essential, are not enough on their own; the implementation capacities of the National Health Security Office also matter.

### Deliberative process

Representation by civil society organizations in multistakeholder governing bodies is essential to sustain transparency and accountability. Allowing civil society to contribute to health policy decisions demonstrates a strong, connected relationship between the state and society.^48^ In Thailand, the relationship has grown out of several opportunities for building networks and has enabled bureaucrats and civil society organizations to share ideas, tactics and resources.^39^ Civil society representatives in the National Health Security Board are well educated and the recommendations they present during board deliberations are based on evidence generated through their networks with research agencies. This evidence-based political culture has evolved gradually since the policy formation phase of the universal coverage scheme.^49^^,^^50^ The continued engagement of civil society organizations in the central decision-making processes of the board has ensured that the scheme developed in ways that benefit citizens. Maintaining the universal coverage scheme requires commitment not only from policy-makers, but also from the civil society organizations^24^ to play active roles in the board.

Box 1 synthesizes challenges and lessons from Thailand’s universal coverage scheme for low- and middle-income countries.

Box 1 **Challenges and lessons from Thailand’s universal health coverage scheme**

**Key challenges**

- Continuity

The current civil society organization cohorts that have been actively engaged since the inception of Thailand’s universal coverage scheme will soon be retiring. Without well planned knowledge transfer and a careful succession plan, civil society contributions to the scheme may be interrupted.

- Transparency

An increasing number of patient groups are supported by the pharmaceutical industry to voice demands for new medicines and technologies that are not currently in the scheme’s benefit package. Although voices from all groups are welcome, the existing transparent process for expanding the benefit package, particularly the use of health technology assessment, must be maintained.

- Accessibility

The platforms to capture citizens’ voices require regular review and strengthening to ensure that they are still effective as intended, that is, to be widely accessible by all people. For example, a survey conducted by a university reported that only 2546 out of 7558 (33.7%) citizens were aware of the telephone helpline in 2018. In addition, the call centre reported that only 11 out of 5248 complaints (0.2%) were about unjustifiable charging by providers in 2018, while the satisfaction survey in the same year showed 73 (3.0%) of 2451 surveyed patients reported being charged by providers.^4^ A constantly low level of complaints may reflect that a helpline may not be the preferred channel for people to voice complaints for which the National Health Security Office needs to test other innovative platforms.

**Key lessons**

- Legislative provisions for voice and accountability

By giving citizens the constitutional right to submit draft bills, the government allowed civil society representatives to insert provisions on voice and accountability into legislative texts that were later adopted under the provisions of the Thailand’s National Health Security Act 2002. Civil society representatives in the parliamentary committee at the second reading of the draft bill seized the opportunity to translate these inspirations into legislative provisions.

- Institutional capacity to implement legislation

Ensuring citizens’ rights to health services requires the office responsible for the scheme to have the necessary implementation capacity. In Thailand, the National Health Security Office needed the capacity to register all 47 million members of the universal coverage scheme and match them with the health-care provider network in the district where they live, and to re-register members to a new network if they moved districts. The full coverage of citizen registration for births and deaths using 13-digit unique national identification numbers and existing extensive geographical coverage of primary health-care services were key enabling factors.

Establishing, sustaining and strengthening the call centre requires continuity of policy and financial support. Timely responses by management to complaints fosters trust among citizens.

Annual public hearings need to be inclusive of citizens, health-care providers, civil society organizations and stakeholders, such as local governments and patient groups. Subsequent policy and management responses are key for building trust in the process and citizens’ ownership of the universal coverage scheme.

Annual public reporting by the office responsible for the scheme (for example, implementation outcomes and performance of the scheme against targets) fosters transparency and increases citizens’ trust in the universal coverage scheme and its management.

## Conclusion

The worldwide governance indicators have not yet been developed to capture the progress of sectoral governance for policy interventions. Despite the overall deteriorating trend of voice and accountability in Thailand’s indicators, and poorly managed public hearings and environment and health impact assessments, the health sector is moving in a more promising direction. Legislative provisions, the nature of the governing body, institutional capacities and deliberative processes have combined to ensure that citizens’ voices are heard, action is taken and the body responsible for the scheme is accountable to both citizens and health-care providers. 
